# Travel history and occupation among diagnosed Cutaneous Leishmaniasis cases in Guyana: A multi-site retrospective case series, 2017–2021

**DOI:** 10.1371/journal.pntd.0014800

**Published:** 2026-10-13

**Authors:** Rajini Kurup, Tiffany Roberts, Jimmy Naipaul, Zeska Williams, Alexus Michael, Annastacia Sampson, Reza Niles-Robin, Rita Persaud, Abilash Nair, Tejroi Naipaul

**Affiliations:** 1 College of Medical Sciences, University of Guyana, Georgetown, Guyana; 2 Caribbean Center for Health and Wellness, Castries, Saint Lucia; 3 Vector Control Services, Ministry of Health, Georgetown, Guyana; 4 Lazaridis School of Business and Economics, Wilfrid Laurier University, Waterloo, Ontario, Canada; 5 Research Department, Joshi Health Inc., Oakville, Ontario, Canada; 6 Office of Research, International Executive School, Strasbourg, France; Marie Adelaide Leprosy Center, PAKISTAN

## Abstract

**Background:**

Cutaneous Leishmaniasis (CL) in Guyana has not been systematically characterised in the peer-reviewed literature for several decades. This study aimed to describe the demographic, occupational, geographic, and clinical profile of diagnosed CL cases at Guyana's principal diagnostic sites, and to test, within this case series, whether field-based occupation is associated with reported interior travel and whether travel history is associated with time to presentation.

**Methodology/Principal Findings:**

Retrospective case series (chart review) of CL diagnoses recorded at three facilities in Georgetown and the interior (2017–2021). Of 223 available case records, a random subsample of 170 was abstracted. Occupation and interior travel were patient-declared; their association was tested directly, then via adjusted logistic regression. Most patients (61.8%) reported interior travel, and 45.3% held field-based occupations, chiefly mining and military. Interior travel was more common among field-based than non-field workers (84.4% vs 43.0%; OR = 7.18 [95% CI, 3.42-15.04]; p < .001), and this association persisted after adjustment for age and sex (OR = 7.08 [3.07-16.30]). Younger age was associated with interior travel (OR = 0.96 [0.94-0.98]; p = .001), whereas sex was not. The sample was predominantly male (81.8%; median age 33 years). Patients with interior travel presented earlier than those without (Mdn = 61 vs 243 days; p < .001).

**Conclusions/Significance:**

Most diagnosed CL cases in this series occurred in people reporting interior travel, predominantly those in field-based occupations, and this travel history was associated with earlier presentation. All participants had CL and there was no comparison group, so these findings describe the case population and associations within it rather than risk factors for acquiring infection; they also rest on a single, retrospectively recorded travel variable. They nonetheless indicate where pre-deployment prevention, occupational screening, and traveller advisories could be focused, and they support a case-control study with validated exposure measurement.

## Author summary

In Guyana, cutaneous leishmaniasis is a skin disease spread by the bite of infected sandflies, which are found mainly in the forested interior of the country. The records of 170 patients diagnosed with this disease at three health facilities between 2017 and 2021 were reviewed to describe who was affected. Most patients had recently travelled into the interior, and this was especially common among miners and soldiers. Younger patients were more likely to have travelled, and those who had travelled sought care much sooner after their skin sore appeared than those who had not. The study included only people who already had the disease, so it cannot show that travel or a particular job causes infection; it shows how these characteristics are distributed among patients. Even so, these findings can help Guyana's health authorities decide where to focus prevention efforts, such as protective measures for workers heading into the interior and faster diagnosis for anyone returning from such trips with a new skin lesion. They also point to the need for studies that compare people with and without the disease.

## Introduction

Leishmaniasis is a vector-borne parasitic disease caused by protozoa of the genus Leishmania, transmitted through the bites of infected female phlebotomine sandflies [1]. Among its clinical manifestations, cutaneous leishmaniasis (CL) is the most prevalent form worldwide, responsible for an estimated 700,000–1,000,000 new cases annually [2,3]. In the Americas, CL is endemic across much of Central and South America, where multiple Leishmania species maintain sylvatic transmission cycles that place forest-exposed individuals at particular risk [4,5].

Guyana presents a distinctive epidemiological context. The country's fewer than 900,000 inhabitants are concentrated along a narrow Atlantic coastal plain [6,7], while the vast interior, encompassing primary tropical rainforest, savannah, and remote hinterland communities, supports artisanal and small-scale gold mining, timber harvesting, and commercial agriculture [8]. These industries generate sustained population movement between comparatively low-exposure coastal environments and high-exposure forested interior settings where phlebotomine sandfly vectors thrive. Guyana's geographic position places it at the intersection of several CL-endemic territories: the interior borders Brazil, a country with one of the highest global CL burdens, while the northwest approaches Venezuela, exposing border communities to epidemiological risk from multiple directions [9].

Studies from neighbouring Brazil, Suriname, and French Guiana have consistently identified gold miners, soldiers, and agricultural workers as the principal high-risk groups for CL acquisition, driven by occupational incursion into forest environments where sandfly vector density is highest [10–17]. More broadly, a systematic review of occupational exposure to leishmaniasis and other vector-borne diseases found soldiers, travellers, migrant workers, miners, and farmers to be the most frequently studied at-risk groups [18]. Yet no systematic, multi-site characterisation of diagnosed CL cases across Guyana's diagnostic infrastructure has been published in the peer-reviewed literature since an isolated outbreak report nearly four decades ago [19]. This gap limits the capacity of public health authorities to identify the populations at greatest risk, design targeted interventions, and allocate resources appropriately within Guyana's national disease control framework.

The present study describes CL cases diagnosed across Guyana's primary diagnostic infrastructure. It had two objectives: (i) to describe the demographic, occupational, geographic, and clinical profile of diagnosed CL cases at Guyana's principal diagnostic sites between 2017 and 2021; and (ii) to test, within this case series, whether field-based occupation is associated with reported interior travel, and whether reported interior travel is associated with time to presentation. It was hypothesised that, among diagnosed cases, patients in field-based occupations would be more likely than those in non-field occupations to report interior travel, and that lesion duration at presentation would differ between travellers and non-travellers. The study includes only people diagnosed with CL and was therefore designed to describe the case population and associations within it, not to estimate the risk of acquiring CL.

## Methods

### Ethical considerations

Ethical approval was granted by the Ministry of Health, Guyana. All patient records were anonymised prior to abstraction using an institutional Identity Protection Code; no patient identifiers appear in any analytic output, and analysis was conducted on aggregate data only. The study constituted a retrospective analysis of routinely collected clinical and laboratory records involving no direct patient contact or intervention. Procedures were conducted in full accordance with the ethical principles of the Declaration of Helsinki and applicable national regulations governing health research in Guyana.

### Study design

This was a retrospective case series based on review of existing clinical and laboratory records covering January 2017 to December 2021, with cross-sectional analysis of the abstracted data; no new data collection or patient contact occurred. All participants had a recorded diagnosis of CL, and no CL-negative comparison group was included. Data were accessed for the purposes of this study between 03/01/2022 and 10/12/2023.

### Study setting and population

The study population was patients receiving a recorded clinical diagnosis of cutaneous leishmaniasis at three facilities constituting the primary institutional infrastructure for CL diagnosis in Guyana: (a) a national reference diagnostic centre in Georgetown (Region 4), receiving referred samples and patients from across all ten administrative regions; (b) a regional general hospital serving a major interior mining and forest community in the northwest (Region 1); and (c) a regional general hospital serving a remote southern border community in the Rupununi (Region 9), adjacent to the international boundary with Brazil. Additional CL diagnoses made at a border-area health facility on the Brazilian side of that boundary were captured where clinical records were available and patients were identifiably presenting from Guyanese territory. Diagnosis was made by the attending clinician at each site according to standard local practice; the specific diagnostic method, which may have included microscopy, serology, clinical criteria, or a combination, was not systematically recorded at the individual-patient level and therefore could not be reported. For the purposes of this study, a case was therefore defined as any patient recorded in a participating facility's laboratory register, microscopy report, or case notification form as having a diagnosis of CL during the study period. The term “diagnosed case” is used throughout to reflect this definition.

Not every diagnosed case at these facilities could be included. A total of 223 cases were available for the study; this figure is the number of records that participating facilities were able to locate, retain in intact form, and share, rather than a complete count of all CL diagnoses managed at these sites over the study period. Facility registers indicate that a larger number of cases had been diagnosed than could ultimately be made available, due to gaps in record-keeping, archival condition, and capacity to support retrieval. The 223 cases available for the study should therefore be read as a lower bound on facility caseload rather than a complete enumeration. Inclusion depended on record availability, so the available cases may differ from all diagnosed cases, for example by over-representing facilities or periods with more complete record-keeping. This potential selection bias arose before, and cannot be corrected by, the random subsampling described below.

Clinical and laboratory records at all three facilities are maintained in paper-based archives rather than an electronic medical record system, meaning that data abstraction required manual retrieval and review of each individual case file on-site. Given the absence of dedicated study funding, abstracting all 223 cases in full was not logistically feasible within the resources available to the study team. Rather than abstracting cases on a convenience basis, which would have risked introducing selection bias into which records were reviewed, a random subsample was therefore drawn to determine which case files would undergo abstraction, intended to minimise the risk that the analytic sample would differ systematically from the available case pool despite the constraint on abstraction capacity. A numbered list of these cases was first compiled from the facility registers and logbooks; 170 case numbers were then selected from this list using SPSS's random case selection function, and only the corresponding physical case files were subsequently retrieved from the archives for abstraction. An a priori power analysis for the primary association (field-based occupation by interior travel; χ² test, 1 df, two-sided α = .05) indicated that 170 cases would provide 97.4% power to detect a medium effect (Cohen's w = 0.3), while remaining within the abstraction capacity available. No cases were individually selected for abstraction by the study team. The flow of cases from the available pool to the analytic sample is shown in Fig 1.

**Fig 1:**
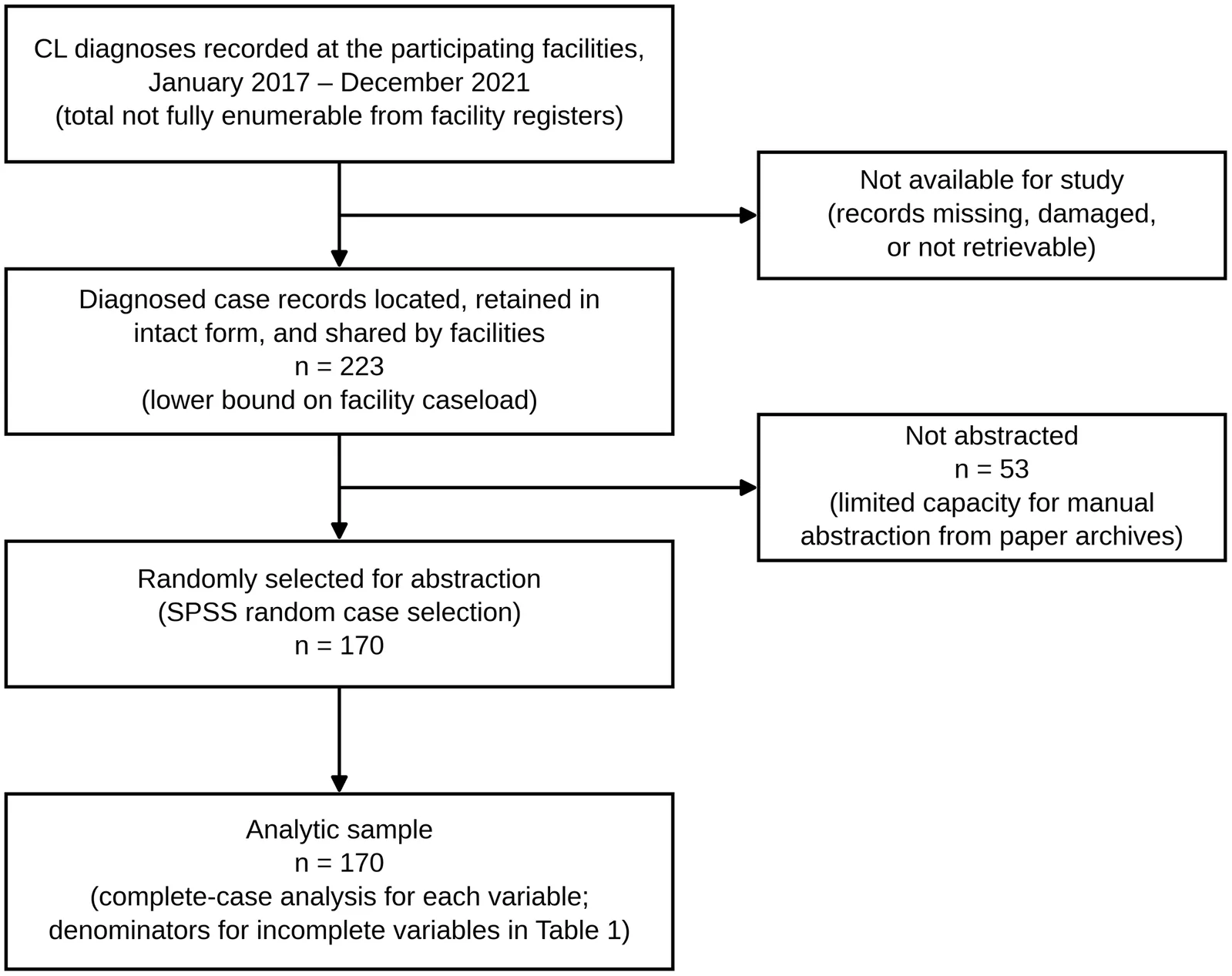
Flow of case ascertainment and sampling. Of the diagnosed CL cases recorded at the participating facilities in 2017–2021, 223 case records could be located, retained in intact form, and shared (a lower bound on facility caseload). A random subsample of 170 was selected for abstraction using SPSS random case selection and formed the analytic sample; the remaining 53 were not abstracted because of limited abstraction capacity. Denominators for variables with incomplete data are given in Table 1.

### Data abstraction

For each of the 170 cases retrieved for abstraction, data were drawn from the laboratory registers, microscopy reports, and case notification forms held at the relevant facility. The primary geographic variable abstracted was the administrative region of patient residence at time of diagnosis, assigned according to Guyana's ten administrative region classification and used to describe the geographic distribution of diagnosed cases. Cases with no recorded residential region (n = 2 in the analytic sample) were retained in overall counts but excluded from regional distribution analyses.

Demographic variables abstracted were age (years), sex, and self-reported ethnic group. Both occupation and interior travel history were patient-declared at the time of presentation, rather than inferred or assigned by the research team. Patients themselves reported their occupation, and these statements were recorded by the attending clinician in the free-text clinical record as given. The research team then classified each patient's declared occupation, a priori, as (i) field-based: occupations characterised by sustained or repeated work activity in forested, agricultural, or open-field environments where contact with phlebotomine sandfly vectors is plausible, specifically miners, soldiers, farmers, lumber jacks, loggers, surveyors, ranchers, community health workers, and government natural resource officers; or (ii) non-field: all remaining occupations, the unemployed, students, and those recording no occupation. Patients with no occupation recorded were therefore classified as non-field rather than treated as missing. This classification was based on the nature and setting of the work itself, independent of whether the patient separately reported travelling to the interior; the two variables were captured and analysed separately for this reason. Interior travel history was based entirely on whether the patient described, or did not describe, an excursion into Guyana's interior beyond their registered administrative region of residence; patients who did not describe such travel were coded as non-travellers. Lesion characteristics abstracted included anatomical location, duration prior to presentation (days), and presence of multiple lesions.

### Data analysis

Data were analysed using IBM SPSS Statistics (Version 29.0; IBM Corp., Armonk, NY) and Python 3.11. The random subsample of 170 cases was drawn using the SPSS random case selection function. Continuous variables are presented as mean (M) and standard deviation (SD) or median (Mdn) and interquartile range (IQR); categorical variables as count and percentage.

Associations between categorical variables were assessed using chi-squared (χ^2^) tests or Fisher's exact test; odds ratios (OR) and 95% confidence intervals (CI) were calculated using the Woolf method. The primary association of interest, between field-based occupational category and interior travel, was tested directly using this chi-squared approach prior to regression modelling. Continuous variables were compared between groups using the Mann-Whitney U test.

Binary logistic regression was then performed with interior travel (yes/no) as the criterion variable and field-based occupational category, age (continuous), and sex as predictors, to determine whether this association remained significant after adjustment for age and sex; model fit was evaluated using the overall model chi-square and Nagelkerke R^2^. Region of residence was examined descriptively rather than entered as a model covariate, both because of its conceptual overlap with facility of presentation and because its ten-plus categories would have strained a model of this sample size.

All hypothesis tests were two-tailed at α = .05. The pre-specified primary analyses were the association between field-based occupation and interior travel (unadjusted and adjusted) and the comparison of lesion duration between travellers and non-travellers. All other comparisons (sex, ethnicity, and region of residence by occupational category or travel status, and comparisons of lesion duration for individual occupational categories against all other patients) were secondary or exploratory. The individual-occupation comparisons of presentation timing were not pre-specified; they were undertaken after the traveller/non-traveller difference in lesion duration was observed.

No correction for multiple comparisons was applied, so secondary and exploratory results are reported as hypothesis-generating, and instances where they would not withstand Bonferroni correction are indicated. Each analysis used all cases with data on the relevant variables (complete-case analysis), and denominators are reported wherever data were missing.

## Results

### Demographic characteristics of the analytic sample

The analytic sample is described below in turn by demographic and clinical characteristics, geographic distribution, occupational composition, the unadjusted association between interior travel and occupation, the same association after adjustment for age and sex, and an exploratory comparison of presentation timing across occupations. The analytic sample of 170 diagnosed CL cases was predominantly male (n = 139; 81.8%), with a mean age of 35.0 years (SD = 14.5; range = 6–86) and a median age of 33 years (IQR = 23–46). The modal age group was 18–34 years (n = 76; 44.7%); 16 participants (9.4%) were younger than 18 years and 28 (16.5%) were aged 50 years or older. Annual case counts in the analytic sample ranged from 29 (2020) to 44 (2019). Data were complete for age, sex, occupational category, and lesion duration (patients with no occupation recorded being classified as non-field; see Methods). Ethnicity was not recorded for 1 patient (0.6%) and region of residence for 2 (1.2%). Information on multiple lesions was available for 128 of 170 patients (75.3%) and on anatomical location for 120 (70.6%), so these characteristics should be interpreted with the extent of missing data in mind (Table 1).

**Table 1:** Demographic, occupational, geographic, and clinical characteristics of the analytic sample (n = 170) of diagnosed Cutaneous Leishmaniasis cases across three diagnostic facilities in Guyana, 2017–2021.

Variable	n	% or M (SD) / Mdn (IQR)
Age (years)		
M (SD)		35.0 (14.5)
Mdn (IQR)		33 (23–46)
Range		6–86
< 18 years	16	9.4
18–34 years	76	44.7
35–49 years	50	29.4
≥ 50 years	28	16.5
Sex		
Male	139	81.8
Female	31	18.2
Ethnicity		
Afro-Guyanese	66	38.8
Mixed Race	40	23.5
Amerindian	34	20.0
Indo-Guyanese	29	17.1
Not recorded	1	0.6
Region of Residence		
Region 4 - Demerara-Mahaica (Georgetown)	55	32.4
Region 7 - Cuyuni-Mazaruni	37	21.8
Region 9 - Upper Takutu–Upper Essequibo (Rupununi)	22	12.9
Region 3 - Essequibo Islands–W. Demerara	13	7.6
Region 10 - Upper Demerara-Berbice (Linden)	9	5.3
Region 8 - Potaro-Siparuni	5	2.9
Regions 1, 2, 5, and 6 combined	27	15.9
Not recorded	2	1.2
Occupational Category		
Field-based occupations	77	45.3
Miners	36	46.8†
Soldiers / military	21	27.3†
Farmers	9	11.7†
Loggers, surveyors, and other field	11	14.3†
Non-field occupations	93	54.7
Interior Travel History		
Travelled to interior region	105	61.8
No travel documented	65	38.2
Lesion Characteristics		
Duration prior to presentation		
Mdn (IQR), days		90 (31–274)
Maximum, days		4,380
Multiple lesions (of n = 128 with data)	43	33.6
Anatomical location (of n = 120 with data)		
Lower limb only	81	67.5
Upper limb only	17	14.2
Trunk only	6	5.0
Face / head only	4	3.3
Generalised	5	4.2
Multiple sites	7	5.8

**Note.** M = mean; SD = standard deviation; Mdn = median; IQR = interquartile range. Percentages may not sum to 100 due to rounding. Field-based occupations include miners, soldiers, farmers, lumber jacks, loggers, surveyors, ranchers, community health workers, and government natural resource officers. Lesion location percentages are of those with a documented anatomical site (n = 120). Data on multiple lesions were available for 128 patients; ethnicity was not recorded for 1 patient and region of residence for 2. †Percentage of the field-based occupational subgroup (n = 77), not of the full analytic sample (n = 170).

The ethnic composition of the sample was Afro-Guyanese (n = 66; 38.8%), Mixed Race (n = 40; 23.5%), Amerindian (n = 34; 20.0%), and Indo-Guyanese (n = 29; 17.1%); ethnicity was not recorded for 1 patient (0.6%). Complete sample characteristics are presented in Table 1.

### Geographic distribution and travel patterns by region of residence

Cases were distributed across all ten of Guyana's administrative regions (Table 1; Fig 2). Region 4 (Georgetown) contributed the largest number of cases (n = 55), reflecting its role as the most populous region and the site of the national reference centre, while interior Regions 7, 8, and 9 together accounted for 38.1% of cases with a recorded region (64 of 168), compared with approximately 8.4% of the national population (73,866 of 878,674 residents in the 2022 census, the census closest to the study period) [20]. Region 4, by contrast, accounted for 32.7% of cases with a recorded region (55 of 168) but 39.6% of the national population [20]. Reported interior travel differed by region of residence. Travel was reported by 42 of 55 Region 4 residents (76.4%) and 12 of 13 Region 3 residents (92.3%). These rates are consistent with many coastal-resident cases having acquired infection during excursions to the interior rather than through local transmission, though this cannot be confirmed without exposure data beyond self-reported travel history. By contrast, 30 of 64 residents of interior Regions 7, 8, and 9 (46.9%) reported travel, consistent with a greater role for exposure near home.

**Fig 2:**
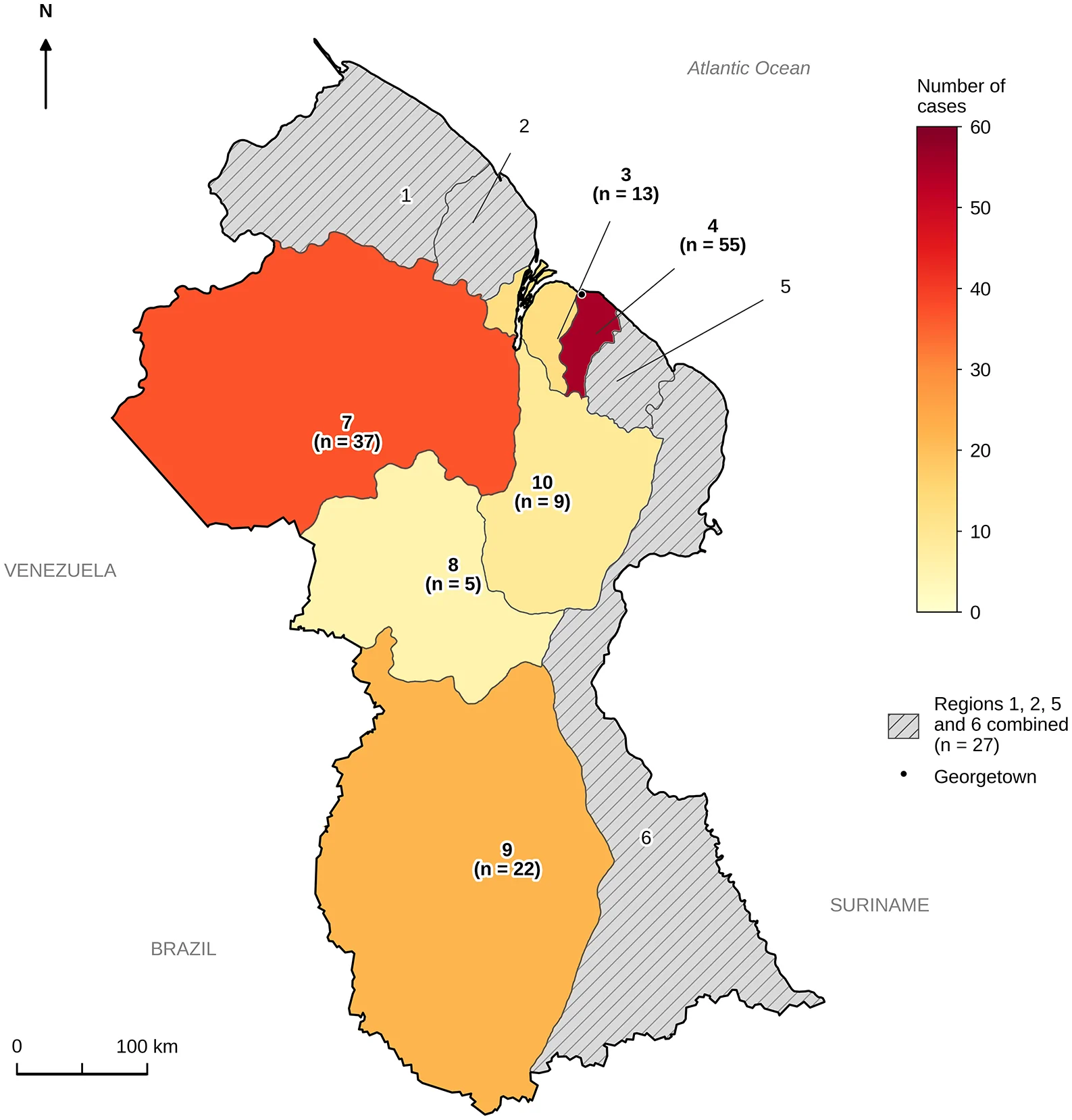
Diagnosed cutaneous leishmaniasis cases by administrative region of residence, Guyana, 2017–2021. Regions are shaded by the number of cases among the 168 patients with a recorded region of residence (region not recorded, n = 2). Regions 1, 2, 5, and 6 are shown as a single combined group (n = 27), as in Table 1. The point marks Georgetown. Base layer: Guyana ADM2 (neighbourhood council) boundaries from geoBoundaries (https://www.geoboundaries.org/api/current/gbOpen/GUY/ADM2/; Runfola et al., PLoS ONE 2020;15(4):e0231866), derived from OCHA FISS and DEVInfo LAC data (https://data.humdata.org/dataset/guyana-administrative-level-0-1-and-2-boundaries) and licensed under CC BY 3.0 IGO (https://creativecommons.org/licenses/by/3.0/igo/). Regional boundaries were derived by merging neighbourhood council units. Map produced by the authors in Python using matplotlib.

### Occupational composition of the sample

Of the 170 cases, 77 (45.3%) were engaged in field-based occupations. Miners were the most represented group (n = 36; 21.2%), followed by soldiers (n = 21; 12.4%) and farmers (n = 9; 5.3%). All 77 patients in field-based occupations were male, compared with 62 of 93 (66.7%) in non-field categories (Fisher's exact p < .001). In an exploratory comparison, Afro-Guyanese patients were more likely to hold field-based occupations (60.6%) than those in other ethnic groups (range = 32.5–38.2%; χ^2^(3) = 10.19, p = .017). This comparison was not pre-specified and would not remain significant after Bonferroni correction for the secondary comparisons made, so it should be regarded as descriptive; it may reflect the demographic composition of Guyana's mining and military workforce. The occupational breakdown, including travel rates, sex distribution, and median lesion duration by category, is presented in Table 2.

**Table 2:** Occupational category, interior travel rate, sex distribution, and lesion duration among the analytic sample (n = 170).

Occupation	n	% of Sample	Travelled to Interior, n (%)	Male sex, n (%)	Lesion Duration, Mdn, days
**Field-based occupations**					
Miner	36	21.2	33 (91.7)	36 (100)	76
Soldier / military	21	12.4	20 (95.2)	21 (100)	30
Farmer	9	5.3	4 (44.4)	9 (100)	138
Logger / lumber jack	4	2.4	2 (50.0)	4 (100)	42
Other field-based	7	4.1	6 (85.7)	7 (100)	60
**Subtotal**	**77**	**45.3**	**65 (84.4)**	**77 (100)**	**61**
**Non-field occupations**					
No occupation	37	21.8	16 (43.2)	24 (64.9)	304
Student	10	5.9	2 (20.0)	9 (90.0)	91
Self-employed	9	5.3	2 (22.2)	4 (44.4)	274
Labourer	5	2.9	3 (60.0)	5 (100)	213
Housewife / domestic	2	1.2	0 (0)	0 (0)	594
Driver	3	1.8	2 (66.7)	3 (100)	121
Other	27	15.9	15 (55.6)	17 (63.0)	60
**Subtotal**	**93**	**54.7**	**40 (43.0)**	**62 (66.7)**	**92**

**Note.** Travel = proportion of each occupational group reporting a history of interior travel. Lesion duration values are medians (days); interquartile ranges are omitted here given small cell sizes within several occupational categories and are reported instead at the aggregate traveller/non-traveller level in Table 3. Several occupational categories (e.g., farmer, n = 9; logger/lumber jack, n = 4; driver, n = 3) are too small to support stable estimates and should be interpreted as descriptive rather than as reliable subgroup comparisons; inference at the level of individual occupations is appropriately limited to the larger miner and soldier/military categories. Other field-based category includes surveyors, ranchers, community health workers, GGMC officers, servicemen, and wardens. Subtotal rows in bold.

### Interior travel and occupation: Unadjusted association

Of the 170 patients, 105 (61.8%) reported travel to interior or hinterland regions prior to presentation. The most frequently cited destinations were Tacama (n = 15), Marudi (n = 6), Ekereku, Mahdia, New River, Omai, and Blackwater/Bartica (n = 3 each), all hinterland locations in Regions 7–10 associated with mining, military operations, or remote forest settlement.

Field-based occupational category was strongly associated with interior travel: 65 of 77 patients with field-based occupations (84.4%) reported travel, compared with 40 of 93 patients with non-field occupations (43.0%; OR = 7.18 [95% CI, 3.42–15.04]; χ^2^(1) = 28.85, p < .001). This shows directly, rather than by inference from occupational title alone, that miners and soldiers in this case series reported interior travel at substantially higher rates than other patients. Farmers, by contrast, reported travel at a rate (4 of 9; 44.4%) similar to that of non-field patients. Military personnel showed the highest travel rate within any occupational group (20 of 21; 95.2%), with Tacama the most commonly cited destination. Miners showed a similarly high travel rate (33 of 36; 91.7%). Travellers were significantly younger than non-travellers (M = 32.3 vs 39.4 years; Mann-Whitney U = 2,512, p = .004). Lesion duration was markedly shorter at presentation among travellers (Mdn = 61 days, IQR = 31–92) than among non-travellers (Mdn = 243 days, IQR = 91–731; Mann-Whitney U = 867, p < .001). Key comparisons between travellers and non-travellers are presented in Table 3.

**Table 3:** Comparison of key characteristics between diagnosed CL patients with and without a history of interior travel (n = 170).

Variable	Travellers (n = 105)	Non-travellers (n = 65)	OR [95% CI]	p
Field-based occupation, n (%)	65 (61.9)	12 (18.5)	7.18 [3.42, 15.04]	<.001**
Male sex, n (%)	94 (89.5)	45 (69.2)	3.80 [1.68, 8.60]	.002**
Age, M (SD), years	32.3 (12.0)	39.4 (16.9)	–	.004†**
Lesion duration, Mdn (IQR), days	61 (31–92)	243 (91–731)	–	<.001†**
Region of residence, n (%)				<.001‡**
Region 4 (Demerara-Mahaica)	42 (40.0)	13 (20.0)	–	
Region 3 (Essequibo Islands–W. Demerara)	12 (11.4)	1 (1.5)	–	
Regions 7, 8, and 9 (interior)	30 (28.6)	34 (52.3)	–	
Other regions or not recorded	21 (20.0)	17 (26.2)	–	

**Note.** OR = odds ratio (Fisher's exact test, Woolf method for 95% CI). †Mann-Whitney U test. OR not applicable (-) for continuous variables. Region percentages are column percentages; ‡χ²(3) test of region distribution by travel status (exploratory; one cell had an expected count of 4.97). ** p < .01.

Patients who did not report interior travel (n = 65) differed from travellers in several respects (Table 3). Just over half (34 of 65; 52.3%) resided in interior Regions 7, 8, or 9, compared with 30 of 105 travellers (28.6%), consistent with a larger share of non-travellers having been exposed near home; a further 13 (20.0%) resided in Region 4. Most non-travellers (53 of 65; 81.5%) held non-field occupations, most commonly no recorded occupation (n = 21), student (n = 8), and self-employed (n = 7), and 20 (30.8%) were female, compared with 11 of 105 travellers (10.5%). Non-travellers were also older and presented after longer lesion durations (Table 3). The records contained no information on peridomestic exposure, housing, or proximity to forest, so the setting in which non-travellers acquired infection could not be determined.

### Interior travel and occupation: Adjusted analysis

Binary logistic regression was conducted with interior travel as the criterion and field-based occupational category, age, and sex as predictors (n = 170). The overall model was statistically significant (χ^2^(3) = 44.98, p < .001; Nagelkerke R^2^ = .316).

Field-based occupational category was the strongest independent correlate of reported interior travel (B = 1.96, SE = 0.43, Wald χ^2^ = 21.14, p < .001; OR = 7.08, 95% CI [3.07, 16.30]), with the association holding at a similar magnitude after adjustment for age and sex (OR = 7.08 vs the unadjusted OR = 7.18), indicating that age and sex account for little of the occupation-travel relationship. Younger age was also a significant independent predictor (B = −0.04, SE = 0.01, Wald χ^2^ = 10.60, p = .001; OR = 0.96, 95% CI [0.94, 0.98]). Male sex did not reach significance after adjustment for occupational category (B = 0.47, Wald χ^2^ = 1.00, p = .317; OR = 1.61, 95% CI [0.64, 4.06]). Full regression results are presented in Table 4.

**Table 4:** Binary logistic regression results using interior travel as the criterion variable (n = 170).

Predictor	B	SE(B)	Wald χ²	p	OR	95% CI [LL, UL]
Intercept	0.83	0.57	2.08	.149	2.29	[0.74, 7.03]
Field-based occupation	1.96**	0.43	21.14	<.001	7.08	[3.07, 16.30]
Age (per year)	−0.04**	0.01	10.60	.001	0.96	[0.94, 0.98]
Male sex	0.47	0.47	1.00	.317	1.61	[0.64, 4.06]
						

**Note.** Model χ^2^(3) = 44.98, p < .001; Nagelkerke R^2^ = .316. B = unstandardized logistic coefficient; SE(B) = standard error; Wald χ^2^ = Wald chi-square statistic; OR = odds ratio; LL = lower limit; UL = upper limit of 95% confidence interval. ** p < .01.

### Exploratory analysis of presentation timing by occupation

Given the magnitude of the travel-related difference in lesion duration, post hoc exploratory comparisons of individual occupational categories against all other patients combined were conducted to assess whether this difference was uniform across field-based occupations. These comparisons were not pre-specified and should be regarded as hypothesis-generating. Soldiers presented with a markedly shorter median lesion duration than the remainder of the sample (Mdn = 31 vs 91 days; Mann-Whitney U = 530.0, p < .001), a difference that would remain significant after Bonferroni correction for the comparisons made. Miners showed no significant difference in presentation timing relative to other patients (Mdn = 76 vs 90 days; Mann-Whitney U = 1283.5, p = .985), despite both groups reporting comparably high travel rates. The remaining occupational categories were too small for meaningful comparison.

## Discussion

In this multi-site series of diagnosed CL cases in Guyana, most patients reported interior travel before presentation, and this was especially common among those in field-based occupations. Among cases, field-based occupation was associated with more than sevenfold higher odds of reported interior travel. This pattern is consistent with the economic geography of the country, in which the extractive and defence industries that employ a large portion of the workforce require sustained presence in forested interior environments where phlebotomine sandfly vectors are endemic. The study includes only people with CL, so these findings describe how travel and occupation are distributed within the case population. They do not show that either raises the risk of acquiring infection, which would require comparison with people without CL. The findings also rest heavily on a single, retrospectively abstracted travel variable and should be read with that measurement constraint in mind (see Limitations). Within those limits, they provide the first multi-site description of these characteristics among diagnosed CL cases in Guyana in several decades, and they indicate where stronger, prospectively validated measurement would be most informative.

The geographic distribution across all ten administrative regions, with interior Regions 7, 8, and 9 accounting for over a third of cases but approximately 8% of the national population, is consistent with ongoing transmission in hinterland communities. Transmission intensity was not directly assessed, however, and the pattern may also reflect regional differences in healthcare access, referral, and record availability. A hinterland focus would be consistent with sandfly ecology: vector density is highest in undisturbed and recently disturbed primary forest [21]. At the same time, the high rate of reported travel among Georgetown residents (76.4%) suggests that coastal urban populations are not insulated from CL. Many may encounter it through the same labour pathways and return to the coast as patients. This dual geography matters for surveillance design, as neither a coastal-only nor an interior-only case-finding strategy would capture the full picture.

The concentration of field-based occupations among male patients (all 77 field-based cases were male) is likely to be less a biological finding than a social one. Mining, military service, and commercial forestry in Guyana are overwhelmingly male-dominated sectors, and the sex ratio among cases is consistent with this. The regression result supports this interpretation: once occupational category was accounted for, male sex was not independently associated with interior travel (OR = 1.61; p = .317). Public health messaging framed around sex as a risk characteristic may therefore be misplaced, and occupational role is likely to be a more useful target. As female participation in mining and community health work grows, any fixed assumption that CL is predominantly a male disease risks generating surveillance blind spots.

A clinically important finding is the fourfold difference in lesion duration at presentation between travellers (Mdn = 61 days) and non-travellers (Mdn = 243 days). Among non-travellers, the median delay exceeded eight months, and the maximum was more than twelve years. This places Guyana within the global pattern of late CL presentation in low- and middle-income settings, where poor disease awareness and the painless, slowly progressive nature of lesions delay diagnosis [22,23]. The earlier presentation of travellers invites two non-exclusive interpretations. First, a discrete travel episode may provide a temporal anchor that strengthens the motivation to seek care. Second, patients in formal field-based settings, particularly military personnel and those employed by mining companies with occupational health infrastructure, may be subject to systematic monitoring that identifies lesions before self-presentation. Either mechanism would point towards the value of post-travel and post-deployment health screening as an early case-detection strategy. In exploratory analyses, the early-presentation pattern was concentrated among soldiers rather than miners. This may suggest that military deployment carries a more structured pathway to early detection than the informal occupational health arrangements typical of artisanal mining, but it is a post hoc observation that warrants targeted investigation. Both interpretations should also be weighed against a measurement caveat. Interior travel was abstracted from existing clinical records and may therefore be documented more completely for patients presenting acutely with a recent, salient exposure event. Part of the duration difference could therefore reflect differential ascertainment of travel rather than health-seeking behaviour alone.

Patients who did not report interior travel (38.2% of the series) warrant separate consideration. Just over half lived in interior Regions 7, 8, or 9, which is consistent with infection acquired in or near their home communities rather than during a discrete excursion. Others, including 13 residents of Region 4, may have made short or routine trips that were not recorded, given that travel was noted only when patients described it. Non-travellers were older, more often female, and less often in field-based work than travellers, and they presented after a median of 243 days. They are therefore the group least likely to be reached by occupational health programmes and the most likely to benefit from wider awareness of CL among communities and primary care providers in endemic areas. The records did not capture household or peridomestic exposures, so where and how non-travellers acquired infection remains an open question for the case-control study proposed in the Limitations.

The prominence of Tacama as a travel destination, cited by 15 patients (8.8% of the analytic sample), deserves attention. This single installation accounted for a discrete, identifiable share of cases in this series, consistent with outbreaks among military personnel at forest training sites in French Guiana and Brazil, and with the high cumulative incidence of CL reported across deployed military populations [12,13,24,25]. The available records do not include all CL cases diagnosed nationally, so the contribution of this site to the national burden cannot be estimated from these data. Operationally, a targeted intervention at Tacama (personal protective equipment, insect repellent protocols, post-deployment dermatological screening) could plausibly reduce cases linked to this site; basic pre-deployment prevention measures markedly reduced CL incidence among Peruvian military trainees in the Amazon [26]. Such an intervention would nonetheless require prospective evaluation at Tacama. The major mining destinations (Marudi, Ekereku, Mahdia, Omai) present a harder problem, as exposure there is distributed across numerous independent operators rather than a single chain of command.

The southern border dimension, reflected in Region 9 cases adjacent to Brazil's Roraima state, raises a broader concern about surveillance adequacy in cross-border transmission. Leishmania does not respect international boundaries; movement of miners, agricultural workers, and community members between Lethem and its Brazilian counterparts may generate bidirectional risk that a single-country system cannot fully capture, underscoring the need for binational surveillance.

### Implications

These findings carry practical implications, organised around travel, occupation, and cross-border movement. For travel, the marked difference in presentation delay between travellers and non-travellers supports a low-cost measure: a standardised advisory, distributed through clinics and community health workers at coastal departure points, urging anyone returning from interior travel to seek prompt evaluation of any new skin lesion, regardless of occupation. Non-travellers and those in non-field occupations presented far later. They are also less likely to be reached by occupationally targeted programmes, so general public awareness campaigns about CL recognition may carry the greatest marginal benefit for this less visible group.

For occupation, the concentration of cases in field-based work, which was the strongest correlate of reported interior travel in this sample, suggests value in embedding CL prevention within existing occupational health frameworks rather than relying on general population messaging alone. For the Guyana Defence Force, this could include integrating pre-deployment CL education and routine post-deployment dermatological screening into standard protocols for personnel assigned to jungle installations, with Tacama a candidate priority site given the number of cases in this series linked to it. For the mining sector, partnerships between public health authorities, the Guyana Geology and Mines Commission, and mining cooperatives could deliver pre-departure briefings, provide personal protective equipment such as insecticide-treated clothing, and establish routine post-return screening for miners working at interior sites [18]. Agricultural extension services represent a parallel, currently untapped channel for reaching farmers with similar messaging, although farmers were few in this series (n = 9) and their travel patterns resembled those of non-field patients. For cross-border movement, the cases from Region 9 underscore the value of integrated surveillance with Brazil, potentially modelled on existing binational arrangements for other vector-borne diseases along the shared frontier, to capture exposure and case data that neither country's health system can fully see in isolation.

### Limitations and future research

The most consequential limitation concerns measurement of the travel variable itself. Interior travel was captured as a binary, self-reported, retrospectively abstracted field with no standardised prompt or recall window, and it structurally under-detects exposure among residents already living in the interior. The central finding rests on this single variable, so its measurement quality is the principal constraint on how much weight the finding can bear; a prospective study using a structured, validated travel-history instrument would substantially strengthen confidence in it. This compounds with a related gap: diagnostic methods were not documented at the individual-patient level, so misclassification with other dermatological conditions cannot be excluded, and non-travellers (who presented with the longest, most ambiguous delays) are plausibly also the group least likely to have received laboratory confirmation, meaning these two sources of uncertainty are not independent.

A further limitation is the absence of a comparison group of CL-negative individuals; because this study describes diagnosed cases alone, the associations reported here characterise correlates of interior travel within the case population rather than risk factors for acquiring CL, which would require a case-control or cohort design. A natural next step would be a facility-based case-control study, comparing diagnosed CL cases with patients attending the same facilities for other conditions, or with community controls from the same regions, using a structured, validated exposure questionnaire covering travel destinations, timing, and duration, occupation, and peridomestic exposures. Parasitological confirmation with species identification (for example, by PCR) in such a study would also reduce diagnostic misclassification. In addition, census data allowed the regional distribution of cases to be compared with the distribution of the population, but no population-level estimate of interior travel or field-based employment in Guyana could be identified. It was therefore not possible to assess whether these characteristics are more common among cases than in the general population; linkage to labour force or mining-registration data would allow such a comparison. Several secondary comparisons were exploratory and not corrected for multiple testing, and some occupational subgroups (for example, farmers, n = 9) were too small for reliable inference. Occupational categories were also grouped by the research team rather than measured directly, introducing a modest further misclassification risk that structured, prospective exposure assessment could resolve. Region of residence was examined descriptively rather than entered into the regression model, for reasons given in Methods, so whether it carries independent predictive value remains untested.

Case ascertainment is a separate concern. The 223 cases available reflect what participating facilities could locate and share, not their full diagnosed caseload, so the true national burden likely exceeds this figure, and the available records may over-represent facilities or periods with more complete record-keeping; the 170-case analytic sample was itself capped by the resources available for manual abstraction from paper-based archives, and the 53 unabstracted cases could not be compared against it on age, sex, or occupation, though random selection was designed to minimise systematic differences. Cross-border case capture from the Brazilian facility could not be independently verified either. These limitations do not alter the descriptive finding that most diagnosed cases in this series reported interior travel, closely linked to field-based occupation, or the association between travel history and earlier presentation; they define the natural next steps for building on these findings.

## Conclusion

In this multi-site series, the great majority of diagnosed CL cases in Guyana occurred in people reporting interior travel, predominantly those in field-based occupations, and travellers presented for care substantially earlier than non-travellers. The study includes only diagnosed cases, so these findings describe the case population rather than establishing risk factors for infection. They nonetheless provide the most detailed recent account of who is diagnosed with CL in Guyana. Travel-focused measures, such as post-travel symptom awareness and prompt evaluation of new skin lesions, may help reduce diagnostic delay, complemented by occupational health measures in the military and mining sectors, which reach the groups that most often reported interior travel. A case-control study with validated exposure measurement is now needed to establish which exposures increase the risk of acquiring CL and to show where prevention is needed most.
