# Correction: Travel history and occupation among diagnosed Cutaneous Leishmaniasis cases in Guyana: A multi-site retrospective case series, 2017–2021

**DOI:** 10.1371/journal.pntd.0014872

**Published:** 2026-11-13

**Authors:** Rajini Kurup, Tiffany Roberts, Jimmy Naipaul, Zeska Williams, Alexus Michael, Annastacia Sampson, Reza Niles-Robin, Rita Persaud, Abilash Nair, Tejroi Naipaul

There is an error in affiliation 3 for authors Annastacia Sampson and Reza Niles-Robin. The correct affiliation 3 is: Neglected Infectious Diseases, Vector Control Services Department, Ministry of Health, Georgetown, Guyana.

The first and third authors, Rajini Kurup and Jimmy Naipaul, should be noted as shared co- corresponding authors on this work. Rajini Kurup’s email address is: rajini.kurup@uog.edu.gy.
