# Supplementary material for: Travel history and occupation among diagnosed Cutaneous Leishmaniasis cases in Guyana: A multi-site retrospective case series, 2017–2021
Source: PLoS Negl Trop Dis. 2026 Oct 13;20(10):e0014800. doi: 10.1371/journal.pntd.0014800 (PMC13660906; doi:10.1371/journal.pntd.0014800)
Supplement: Supplementary file 1 [file pntd.0014800.s002.docx]

**Response Document**

Dear Editor and Reviewers,

Thank you sincerely for the time and care you devoted to reviewing our manuscript, now titled “Travel History and Occupation Among Diagnosed Cutaneous Leishmaniasis Cases in Guyana: A Multi-Site Retrospective Case Series, 2017–2021”. Your thoughtful and constructive feedback has genuinely elevated the paper. Your guidance on aligning the framing with the case-only design, in particular, has made the manuscript clearer, more rigorous, and more useful to readers, and we are very grateful for it.

We have addressed each comment below. All changes are shown in the file labelled “Revised Manuscript with Track Changes”, and a clean copy is provided as “Manuscript”.

**Reviewer 1**

Comment 1 (Methods): They tell you what they're studying, but never write out a clean, testable hypothesis. You have to infer it.

Response: Thank you for this helpful point. We agree that the hypothesis should be stated explicitly rather than left for the reader to infer. The final paragraph of the Introduction now sets out two objectives and a testable hypothesis: “It had two objectives: (i) to describe the demographic, occupational, geographic, and clinical profile of diagnosed CL cases at Guyana's principal diagnostic sites between 2017 and 2021; and (ii) to test, within this case series, whether field-based occupation is associated with reported interior travel, and whether reported interior travel is associated with time to presentation.” This is followed by: “It was hypothesised that, among diagnosed cases, patients in field-based occupations would be more likely than those in non-field occupations to report interior travel, and that lesion duration at presentation would differ between travellers and non-travellers.” The Background section of the Abstract has been revised to match.

Comment 2 (Methods): Appropriate for a descriptive/associative aim, but not for the causal or "risk factor" framing the paper sometimes uses (abstract: "risk pathway"; Discussion: "occupation raised the odds"). This is a case-only cross-sectional review, there is no CL-negative comparison group, so nothing here establishes risk of acquiring CL, only correlates of travel history within confirmed cases. The authors do acknowledge this in Limitations, which is good, but the language elsewhere in the abstract/discussion should be tightened to avoid implying risk-factor inference the design can't support.

Response: Thank you for this important observation, with which we fully agree. You are right that the Limitations section acknowledged the case-only design while other parts of the paper did not reflect it, and we have now brought the whole manuscript into line. We removed risk-factor language throughout, including “risk pathway” from the Abstract and “raised the odds” from the Discussion, and we rewrote the Abstract Conclusions, the Author Summary, the opening of the Discussion, the Implications, and the Conclusion. The Study Design now states that the study is a retrospective case series with no CL-negative comparison group. The Discussion now reads: “The study includes only people with CL, so these findings describe how travel and occupation are distributed within the case population. They do not show that either raises the risk of acquiring infection, which would require comparison with people without CL.” Where the odds ratio is reported, it is now described as an association with reported travel among cases.

Comment 3 (Methods): Reasonably well described, but two soft spots: (a) "confirmed case" isn't rigorously defined since they didn't record how each diagnosis was made, and (b) the 223 cases are just whatever records the facilities happened to still have (not all diagnosed cases) so there's selection bias baked in before their random subsampling even starts

Response: Thank you for identifying these two points. (a) We agree that “confirmed” overstated what the records allow, since the diagnostic method was not recorded for each patient. We now use the term “diagnosed case” throughout, including in the title, and have added a case definition to the Methods: “For the purposes of this study, a case was therefore defined as any patient recorded in a participating facility's laboratory register, microscopy report, or case notification form as having a diagnosis of CL during the study period.” (b) We also agree that selection bias arises before the random subsampling. The Methods now state: “Inclusion depended on record availability, so the available cases may differ from all diagnosed cases, for example by over-representing facilities or periods with more complete record-keeping.” This point is repeated in the Limitations.

Comment 4 (Methods): The main analysis (170 patients) is big enough. But they just assert a power calculation without showing the numbers behind it. Also, some occupation subgroups (like farmers, n=9) are way too small to draw real conclusions from, which the authors partly admit.

Response: Thank you for this comment. The Methods now report the parameters of the a priori power calculation: for the primary association between field-based occupation and interior travel (χ² test, 1 df, two-sided α = .05), “170 cases would provide 97.4% power to detect a medium effect (Cohen's w = 0.3)”. We also agree about the small subgroups. Farmers are no longer grouped with miners and soldiers in the Abstract or Results; the Results now note that farmers reported interior travel at a rate (4 of 9; 44.4%) similar to that of non-field patients, and the Implications and Limitations now note that farmers were few and that some occupational subgroups were too small for reliable inference.

Comment 5 (Methods): Broadly yes and appropriately matched to data type. However, no correction for multiple comparisons across the numerous subgroup tests (ethnicity × occupation, individual-occupation-vs-rest duration comparisons, per-region travel rates). Calling the occupation-timing analysis "exploratory" partially addresses this, but the ethnicity–occupation χ² and some other tests are presented without that caveat and read as confirmatory.

Response: Thank you for raising this; we agree. The Data Analysis section now distinguishes the pre-specified primary analyses (the association between field-based occupation and interior travel, and the comparison of lesion duration between travellers and non-travellers) from secondary and exploratory comparisons, and states: “No correction for multiple comparisons was applied, so secondary and exploratory results are reported as hypothesis-generating, and instances where they would not withstand Bonferroni correction are indicated.” In the Results, the ethnicity by occupation comparison (p = .017) is now labelled exploratory, with a note that it would not remain significant after Bonferroni correction, and we note that the soldier comparison of presentation timing (p < .001) would remain significant. The Limitations now also mention uncorrected secondary testing.

Comment 6 (Results): Mostly yes. They said they'd do χ²/Fisher's exact first, then adjusted logistic regression, then look at duration, and that's the order the Results follow. One thing that sneaks in unannounced: the soldier-vs-miner presentation-timing breakdown is introduced as "exploratory" in the Results with no mention in Methods that this comparison was planned.

Response: Thank you for noticing this. You are correct that this comparison was not planned in advance. The Data Analysis section now states that the individual-occupation comparisons of presentation timing “were not pre-specified; they were undertaken after the traveller/non-traveller difference in lesion duration was observed.” The corresponding Results section describes them as post hoc and hypothesis-generating, and the Discussion refers to the soldier and miner contrast as a post hoc observation that warrants targeted investigation.

Comment 7 (Results): Largely yes (numbers, CIs, and p-values are given consistently, and Tables 1-4 back up the text). A few gaps: they never report how many patients had missing occupation or ethnicity data (they do this for region, lesion location, and multiple lesions, but not everywhere). Also the geographic-travel-by-region numbers (76.4% Region 4, 92.3% Region 3, 46.9% interior) might be worth pulling into Table 1 or its own table since they're used to support a Discussion claim.

Response: Thank you for these helpful suggestions. The first paragraph of the Results now summarises the completeness of each variable: data were complete for age, sex, occupational category, and lesion duration; ethnicity was not recorded for 1 patient (0.6%) and region of residence for 2 (1.2%). Ethnicity “Not recorded” is also now shown as a row in Table 1. We have clarified that patients with no occupation recorded were classified as non-field under our a priori scheme rather than treated as missing. For the regional travel rates, the Results now give the numerators and denominators (42 of 55 in Region 4; 12 of 13 in Region 3; 30 of 64 in Regions 7, 8, and 9), and we have added region of residence rows to Table 3, which compares travellers with non-travellers, together with an exploratory χ² test (χ²(3) = 16.79, p < .001). We placed these rows in Table 3 because it already compares the two groups, and this also helps address Reviewer 3's request for more information on non-travellers.

Comment 8 (Results): Tables are decent: well-labeled, footnoted where cell sizes are small. One issue: there's no actual figure, no map of case distribution by region, no flowchart of case ascertainment (223 → 170 → analytic sample), which would help a reader follow the sampling logic a lot faster than the prose does.

Response: Thank you for this excellent suggestion, which we have taken up in full. We have added two figures. Fig 1 is a flow diagram of case ascertainment, showing the facility records, the 223 available case records, the 170 randomly selected for abstraction, and the analytic sample; it is cited in the Methods. Fig 2 is a map showing the number of cases by administrative region of residence; it is cited in the Results alongside Table 1. So that the table and map agree, Region 8 (n = 5) is now reported separately in Table 1, while Regions 1, 2, 5, and 6 remain combined as in the original table.

Comment 9 (Conclusions): Mostly, yes, with one recurring overreach. The data do support "interior travel is common and strongly tied to field-based work, and travellers present sooner." But the paper keeps sliding into risk-factor language ("occupation raised the odds," "risk pathway") when all they actually have is an association among existing cases, no comparison to people without CL.

Response: Thank you; we agree, and we appreciate you highlighting this. Please see our response to Comment 2. The Conclusion now reads, in part: “In this multi-site series, the great majority of diagnosed CL cases in Guyana occurred in people reporting interior travel, predominantly those in field-based occupations, and travellers presented for care substantially earlier than non-travellers.” It continues: “The study includes only diagnosed cases, so these findings describe the case population rather than establishing risk factors for infection.” We have also revised the final sentence of the Limitations, which previously stated that travel was strongly associated with CL diagnosis.

Comment 10 (Conclusions): Yes very clearly, no comment.

Response: Thank you very much for this kind comment.

Comment 11 (Conclusions): Somewhat. They don't say much about what specific next study would close the gap (e.g., a case-control design, a validated travel-history tool), beyond a general "future research should" nod in the limitations.

Response: Thank you for this valuable suggestion. We have added a specific proposal to the Limitations and Future Research section: “A natural next step would be a facility-based case-control study, comparing diagnosed CL cases with patients attending the same facilities for other conditions, or with community controls from the same regions, using a structured, validated exposure questionnaire covering travel destinations, timing, and duration, occupation, and peridomestic exposures.” We also note that parasitological confirmation with species identification in such a study would reduce diagnostic misclassification. The Abstract and the Conclusion now also identify a case-control study with validated exposure measurement as the next step.

Comment 12 (Conclusions): Yes, this is well done.

Response: Thank you, we are very grateful for this encouraging feedback.

Comment 13 (Editorial and data presentation): Some of the wording in the Discussion could be a little more cautious. For example, the statement that the geographic distribution “speaks to transmission intensity in hinterland communities rather than mere healthcare access patterns” feels too strong, as transmission intensity was not directly assessed.

Response: Thank you; we agree that this wording was too strong. The sentence now describes the distribution as consistent with ongoing transmission in hinterland communities, and continues: “Transmission intensity was not directly assessed, however, and the pattern may also reflect regional differences in healthcare access, referral, and record availability.”

Comment 14 (Editorial and data presentation): Similarly, referring to Tacama's contribution to the “national CL burden” may be an overstatement given that the available cases do not represent all CL cases diagnosed nationally.

Response: Thank you; we agree. The Discussion now refers to the cases in this series linked to Tacama and adds: “The available records do not include all CL cases diagnosed nationally, so the contribution of this site to the national burden cannot be estimated from these data.” The corresponding phrase in the Implications has also been revised.

Comment 15 (Editorial and data presentation): It would also help to make the missing clinical data a bit more visible. For example, information on multiple lesions was available for 128 of 170 patients and anatomical location for 120 of 170. Although these denominators are reported in Table 1, briefly mentioning the extent of missing data in the Results would make these findings easier to interpret.

Response: Thank you for this helpful suggestion. The first paragraph of the Results now reports the extent of missing data for each variable, including multiple lesions (available for 128 of 170 patients; 75.3%) and anatomical location (120 of 170; 70.6%), and notes that these characteristics should be interpreted with the extent of missing data in mind. The note to Table 1 has also been updated.

Comment 16 (Editorial and data presentation): Finally, some parts of the Discussion contain quite long sentences with several interpretations and recommendations combined. Breaking these up, particularly in the sections on presentation delay and occupational exposure, would improve readability.

Response: Thank you; we agree that readability could be improved. We have broken up long sentences throughout the Discussion, particularly in the paragraphs on presentation delay, sex and occupation, geographic distribution, and the Implications. We have also divided the Data Analysis section of the Methods into shorter paragraphs.

Comment 17 (Summary and general comments): This study provides useful descriptive data on cutaneous leishmaniasis in Guyana, where recent epidemiological evidence is limited. The multi-site approach and assessment of both interior travel and occupation are strengths. The main weakness is the retrospective, case-only design, which limits the conclusions to associations within confirmed CL cases and does not support broader risk-factor inference. Overall, the study is relevant and well presented, but some conclusions should be more cautiously framed to reflect these limitations.

Response: Thank you for this generous and thoughtful summary. We are glad the study's contribution and strengths came through, and we are grateful for your guidance on the framing. As described above, we have revised the conclusions throughout so that they reflect the case-only design.

**Reviewer 2**

Comment 1: The aim (lines in Introduction, final paragraph; repeated in the Abstract Background) is stated as a descriptive purpose rather than a testable hypothesis. More importantly, the aim as written — to establish “whether the disease burden is disproportionately concentrated among travellers” — is not answerable by this design (see 2). I would suggest restating the objectives as two explicit, design-matched questions, e.g.: (i) to describe the demographic, occupational, geographic and clinical profile of diagnosed CL cases at Guyana’s principal diagnostic sites, 2017–2021; and (ii) to test, within this case series, whether field-based occupation is associated with reported interior travel and with time to presentation. Both are well supported by the data.

Response: Thank you for this very helpful suggestion, which we have adopted. The final paragraph of the Introduction and the Abstract Background now state the two objectives in essentially the form you proposed, followed by an explicit hypothesis (quoted in our response to Reviewer 1, Comment 1). We framed the second part of objective (ii) as the association between reported interior travel and time to presentation, since this is the comparison reported in the primary analysis. The Introduction also now states that the study was designed to describe the case population and associations within it, not to estimate the risk of acquiring CL.

Comment 2: This is a case-only series: every participant has CL. Without a comparison group, and without an external estimate of how common interior travel and field-based work are in the Guyanese population, the proportion of cases reporting travel (61.8%) cannot establish that burden is “concentrated” among travellers, nor that travel is the “proximate exposure”. What the data do support is a description of the case population and an internal association between occupation and travel among cases. The authors state this clearly and fairly in Limitations (paragraph 2) — the problem is that the title, Abstract Conclusions, Discussion opening sentence (“Cutaneous leishmaniasis in Guyana is, at its core, a travel-associated disease burden”), and the Conclusion (“reframes cutaneous leishmaniasis in Guyana as a disease structured primarily by interior travel”) all assert the causal reading that the Limitations section disclaims. I would ask the authors to bring the whole manuscript into line with the Limitations paragraph. Concretely: retitle along the lines of “Travel history and occupation among diagnosed cutaneous leishmaniasis cases in Guyana: a multi-site retrospective series, 2017–2021”, and rephrase the claim to something like “the great majority of diagnosed cases occurred in people reporting interior travel, predominantly in field-based occupations”. If any national or regional estimate of interior travel prevalence exists (census, labour force survey, GGMC registration data), even a rough comparison would materially strengthen the “concentration” argument and would be worth adding.

Response: Thank you for this clear and constructive comment. We agree with your assessment and have made each of the changes you suggested. The title now reads “Travel History and Occupation Among Diagnosed Cutaneous Leishmaniasis Cases in Guyana: A Multi-Site Retrospective Case Series, 2017–2021”. The Abstract Conclusions now state that “Most diagnosed CL cases in this series occurred in people reporting interior travel, predominantly those in field-based occupations”, and that the findings describe the case population rather than risk factors for acquiring infection. In the Discussion, the sentence describing CL as “at its core, a travel-associated disease burden” and the references to travel as the “proximate exposure” have been removed; the Discussion now opens: “In this multi-site series of diagnosed CL cases in Guyana, most patients reported interior travel before presentation, and this was especially common among those in field-based occupations.” In the Conclusion, the sentence beginning “This study reframes cutaneous leishmaniasis in Guyana” has been replaced with wording closely following your suggestion (quoted in our response to Reviewer 1, Comment 9). Similar language has also been removed from the Author Summary and the Implications.

We also appreciated your suggestion of an external comparison and have added one using the 2022 national census, the census closest to the study period (new reference 19). Interior Regions 7, 8, and 9 accounted for 38.1% of cases with a recorded region but approximately 8.4% of the national population (73,866 of 878,674 residents), whereas Region 4 accounted for 32.7% of cases but 39.6% of the population. This comparison is reported in the Results and referred to in the Discussion. As it relates to where cases lived rather than to travel, we were not able to extend it to interior travel itself: we could not identify a population-level estimate of interior travel, or of employment in mining, forestry, or the military, for the study period. We have therefore noted this in the Limitations, together with the suggestion that linkage to labour force or mining-registration data would allow such a comparison in future work.

**Reviewer 3**

Comment 1: It is an interesting report on cutaneous leishmaniasis situation in Guyana. There is a good data on travel transmission of the disease within the country. But a consistent part of the analysed patients did not travel. We have no epidemiologic informations on these patients. The authors could detail the epidemiology of non-travellers patients

Response: Thank you for this thoughtful comment. We agree that the 65 patients who did not report interior travel deserve more attention, and we have addressed this in three ways. First, we added a paragraph to the Results describing this group: just over half (34 of 65; 52.3%) resided in interior Regions 7, 8, or 9, compared with 30 of 105 travellers (28.6%); most (53 of 65; 81.5%) held non-field occupations; 20 (30.8%) were female, compared with 11 of 105 travellers (10.5%); and they were older and presented after longer lesion durations. Second, we added region of residence rows to Table 3 so that travellers and non-travellers can be compared directly. Third, we added a paragraph to the Discussion interpreting these findings. It notes that the residence pattern is consistent with infection acquired in or near home communities, that some coastal non-travellers may have made short trips that were not recorded, and that their later presentation (median 243 days) makes them the group most likely to benefit from wider awareness of CL in endemic communities and primary care. The records did not contain information on household or peridomestic exposures, so where non-travellers acquired infection could not be determined; we state this in the Results and Discussion, and peridomestic exposures are included among the variables proposed for the future case-control study.

Additional changes

In revising the manuscript, we also made the following changes, all of which are shown in tracked changes. The statement that the sample's ethnic composition reflected Guyana's national demographic profile has been removed, as Indo-Guyanese form the largest ethnic group nationally but the smallest in this sample. In the Discussion, the sentence on military transmission at Tacama previously cited sources that did not match the claim; it now cites a systematic review of leishmaniasis in deployed military populations (reference 12), the French Guiana outbreak (reference 13), and two studies of CL at a military training centre in Pernambuco, Brazil (references 23 and 24). Following the addition of the census reference (reference 19), the former references 19 to 23 have been renumbered. Author affiliations have also been updated.

Thank you once again for your generous and insightful feedback. We hope the revised manuscript meets your expectations, and we look forward to hearing from you.
