# Supplementary material for: Travel history and occupation among diagnosed Cutaneous Leishmaniasis cases in Guyana: A multi-site retrospective case series, 2017–2021
Source: PLoS Negl Trop Dis. 2026 Oct 13;20(10):e0014800. doi: 10.1371/journal.pntd.0014800 (PMC13660906; doi:10.1371/journal.pntd.0014800)
Supplement: Peer review history

# Peer review: Decision Letter 0

**DOI:** 10.1371/journal.pntd.0014800.r001

**Authors:** Claudia Brodskyn, Anil Fastenau

22 Sep 2026

Response to Reviewers Revised Manuscript with Track Changes Manuscript

As the corresponding author, your ORCID iD is verified in the submission system and will appear in the published article. PLOS supports the use of ORCID, and we encourage all coauthors to register for an ORCID iD and use it as well. Please encourage your coauthors to verify their ORCID iD within the submission system before final acceptance, as unverified ORCID iDs will not appear in the published article. Only the individual author can complete the verification step; PLOS staff cannot verify ORCID iDs on behalf of authors.

We look forward to receiving your revised manuscript.

Shaden Kamhawi

co-Editor-in-Chief

PLOS Neglected Tropical Diseases

orcid.org/0000-0003-4304-636XX

Paul Brindley

co-Editor-in-Chief

PLOS Neglected Tropical Diseases

orcid.org/0000-0003-1765-0002

**Journal Requirements:**

1) We note that you have indicated that there are restrictions to data sharing for this study. PLOS only allows data to be available upon request if there are legal or ethical restrictions on sharing data publicly. For more information on unacceptable data access restrictions, please see

https://journals.plos.org/plosntds/s/data-availability#loc-unacceptable-data-access-restrictions

Before we proceed with your manuscript, please address the following prompts:

a) If there are ethical or legal restrictions on sharing a de-identified data set, please explain them in detail (e.g., data contain potentially identifying or sensitive patient information, data are owned by a third-party organization, etc.) and who has imposed them (e.g., a Research Ethics Committee or Institutional Review Board, etc.). Please also provide contact information for a data access committee, ethics committee, or other institutional body to which data requests may be sent.

b) If there are no restrictions, please upload the minimal anonymized data set necessary to replicate your study findings to a stable, public repository and provide us with the relevant URLs, DOIs, or accession numbers. For a list of recommended repositories, please see

https://journals.plos.org/plosone/s/recommended-repositories. You also have the option of uploading the data as Supporting Information files, but we would recommend depositing data directly to a data repository if possible.

We will update your Data Availability statement on your behalf to reflect the information you provide.

**Reviewers' comments:**

**Key Review Criteria Required for Acceptance?**

As you describe the new analyses required for acceptance, please consider the following:

**Methods**

-Are the objectives of the study clearly articulated with a clear testable hypothesis stated?

-Is the study design appropriate to address the stated objectives?

-Is the population clearly described and appropriate for the hypothesis being tested?

-Is the sample size sufficient to ensure adequate power to address the hypothesis being tested?

-Were correct statistical analysis used to support conclusions?

-Are there concerns about ethical or regulatory requirements being met?

Reviewer #1: 1. They tell you what they're studying, but never write out a clean, testable hypothesis. You have to infer it.

2. Appropriate for a descriptive/associative aim, but not for the causal or "risk factor" framing the paper sometimes uses (abstract: "risk pathway"; Discussion: "occupation raised the odds"). This is a case-only cross-sectional review, there is no CL-negative comparison group, so nothing here establishes risk of acquiring CL, only correlates of travel history within confirmed cases. The authors do acknowledge this in Limitations, which is good, but the language elsewhere in the abstract/discussion should be tightened to avoid implying risk-factor inference the design can't support.

3. Reasonably well described, but two soft spots: (a) "confirmed case" isn't rigorously defined since they didn't record how each diagnosis was made, and (b) the 223 cases are just whatever records the facilities happened to still have (not all diagnosed cases) so there's selection bias baked in before their random subsampling even starts

4. The main analysis (170 patients) is big enough. But they just assert a power calculation without showing the numbers behind it. Also, some occupation subgroups (like farmers, n=9) are way too small to draw real conclusions from, which the authors partly admit.

5. Broadly yes and appropriately matched to data type. However, no correction for multiple comparisons across the numerous subgroup tests (ethnicity × occupation, individual-occupation-vs-rest duration comparisons, per-region travel rates). Calling the occupation-timing analysis "exploratory" partially addresses this, but the ethnicity–occupation χ² and some other tests are presented without that caveat and read as confirmatory.

Reviewer #2: The aim (lines in Introduction, final paragraph; repeated in the Abstract Background) is stated as a descriptive purpose rather than a testable hypothesis. More importantly, the aim as written — to establish “whether the disease burden is disproportionately concentrated among travellers” — is not answerable by this design (see 2). I would suggest restating the objectives as two explicit, design-matched questions, e.g.: (i) to describe the demographic, occupational, geographic and clinical profile of diagnosed CL cases at Guyana’s principal diagnostic sites, 2017–2021; and (ii) to test, within this case series, whether field-based occupation is associated with reported interior travel and with time to presentation. Both are well supported by the data.

This is a case-only series: every participant has CL. Without a comparison group, and without an external estimate of how common interior travel and field-based work are in the Guyanese population, the proportion of cases reporting travel (61.8%) cannot establish that burden is “concentrated” among travellers, nor that travel is the “proximate exposure”. What the data do support is a description of the case population and an internal association between occupation and travel among cases. The authors state this clearly and fairly in Limitations (paragraph 2) — the problem is that the title, Abstract Conclusions, Discussion opening sentence (“Cutaneous leishmaniasis in Guyana is, at its core, a travel-associated disease burden”), and the Conclusion (“reframes cutaneous leishmaniasis in Guyana as a disease structured primarily by interior travel”) all assert the causal reading that the Limitations section disclaims. I would ask the authors to bring the whole manuscript into line with the Limitations paragraph. Concretely: retitle along the lines of “Travel history and occupation among diagnosed cutaneous leishmaniasis cases in Guyana: a multi-site retrospective series, 2017–2021”, and rephrase the claim to something like “the great majority of diagnosed cases occurred in people reporting interior travel, predominantly in field-based occupations”. If any national or regional estimate of interior travel prevalence exists (census, labour force survey, GGMC registration data), even a rough comparison would materially strengthen the “concentration” argument and would be worth adding.

Reviewer #3: Yes

**********

**Results**

-Does the analysis presented match the analysis plan?

-Are the results clearly and completely presented?

-Are the figures (Tables, Images) of sufficient quality for clarity?

Reviewer #1: 1. Mostly yes. They said they'd do χ²/Fisher's exact first, then adjusted logistic regression, then look at duration, and that's the order the Results follow. One thing that sneaks in unannounced: the soldier-vs-miner presentation-timing breakdown is introduced as "exploratory" in the Results with no mention in Methods that this comparison was planned.

2. Largely yes (numbers, CIs, and p-values are given consistently, and Tables 1-4 back up the text). A few gaps: they never report how many patients had missing occupation or ethnicity data (they do this for region, lesion location, and multiple lesions, but not everywhere). Also the geographic-travel-by-region numbers (76.4% Region 4, 92.3% Region 3, 46.9% interior) might be worth pulling into Table 1 or its own table since they're used to support a Discussion claim.

3. Tables are decent: well-labeled, footnoted where cell sizes are small. One issue: there's no actual figure, no map of case distribution by region, no flowchart of case ascertainment (223 → 170 → analytic sample), which would help a reader follow the sampling logic a lot faster than the prose does.

Reviewer #2: (No Response)

Reviewer #3: Yes

**********

**Conclusions**

-Are the conclusions supported by the data presented?

-Are the limitations of analysis clearly described?

-Do the authors discuss how these data can be helpful to advance our understanding of the topic under study?

-Is public health relevance addressed?

Reviewer #1: 1. Mostly, yes, with one recurring overreach. The data do support "interior travel is common and strongly tied to field-based work, and travellers present sooner." But the paper keeps sliding into risk-factor language ("occupation raised the odds," "risk pathway") when all they actually have is an association among existing cases, no comparison to people without CL.

2. Yes very clearly, no comment.

3. Somewhat. They don't say much about what specific next study would close the gap (e.g., a case-control design, a validated travel-history tool), beyond a general "future research should" nod in the limitations.

4. Yes, this is well done.

Reviewer #2: (No Response)

Reviewer #3: Yes

**********

**Editorial and Data Presentation Modifications?**

Use this section for editorial suggestions as well as relatively minor modifications of existing data that would enhance clarity. If the only modifications needed are minor and/or editorial, you may wish to recommend “Minor Revision” or “Accept”.

Reviewer #1: The manuscript is generally clear and easy to follow, but I have a few minor suggestions that could improve clarity.

Some of the wording in the Discussion could be a little more cautious. For example, the statement that the geographic distribution “speaks to transmission intensity in hinterland communities rather than mere healthcare access patterns” feels too strong, as transmission intensity was not directly assessed. Similarly, referring to Tacama’s contribution to the “national CL burden” may be an overstatement given that the available cases do not represent all CL cases diagnosed nationally.

It would also help to make the missing clinical data a bit more visible. For example, information on multiple lesions was available for 128 of 170 patients and anatomical location for 120 of 170. Although these denominators are reported in Table 1, briefly mentioning the extent of missing data in the Results would make these findings easier to interpret.

Finally, some parts of the Discussion contain quite long sentences with several interpretations and recommendations combined. Breaking these up, particularly in the sections on presentation delay and occupational exposure, would improve readability.

Reviewer #2: (No Response)

Reviewer #3: (No Response)

**********

**Summary and General Comments**

Use this section to provide overall comments, discuss strengths/weaknesses of the study, novelty, significance, general execution and scholarship. You may also include additional comments for the author, including concerns about dual publication, research ethics, or publication ethics. If requesting major revision, please articulate the new experiments that are needed.

Reviewer #1: This study provides useful descriptive data on cutaneous leishmaniasis in Guyana, where recent epidemiological evidence is limited. The multi-site approach and assessment of both interior travel and occupation are strengths. The main weakness is the retrospective, case-only design, which limits the conclusions to associations within confirmed CL cases and does not support broader risk-factor inference. Overall, the study is relevant and well presented, but some conclusions should be more cautiously framed to reflect these limitations.

Reviewer #2: (No Response)

Reviewer #3: It is an interesting report on cutaneous leishmaniasis situation in Guyana. There is a good data on travel transmission of the disease within the country. But a consistent part of the analysed patients did not travel. We have no epidemiologic informations on these patients. The authors could detail the epidemiology of non-travellers patients

**********

PLOS authors have the option to publish the peer review history of their article (what does this mean?). If published, this will include your full peer review and any attached files.

If you choose “no”, your identity will remain anonymous but your review may still be made public.

**Do you want your identity to be public for this peer review?** For information about this choice, including consent withdrawal, please see our Privacy Policy.

Reviewer #1: No

Reviewer #2: No

Reviewer #3: **Yes:**  Mourad MOKNI

**Figure resubmission**

**Reproducibility:** -->To enhance the reproducibility of your results, we recommend that authors of applicable studies deposit laboratory protocols in protocols.io, where a protocol can be assigned its own identifier (DOI) such that it can be cited independently in the future. Additionally, PLOS ONE offers an option to publish peer-reviewed clinical study protocols. Read more information on sharing protocols at https://plos.org/protocols?utm_medium=editorial-email&utm_source=authorletters&utm_campaign=protocols-->?>

# Author comment: Author response to Decision Letter 1

**DOI:** 10.1371/journal.pntd.0014800.r002

24 Sep 2026

Attachment Submitted filename: Response to Reviewers.docx

# Editor report: Decision Letter 1

**DOI:** 10.1371/journal.pntd.0014800.r003

**Authors:** Claudia Brodskyn, Anil Fastenau

27 Sep 2026

Dear Dr Naipaul,

We are pleased to inform you that your manuscript 'Travel History and Occupation Among Diagnosed Cutaneous Leishmaniasis Cases in Guyana: A Multi-Site Retrospective Case Series, 2017–2021' has been provisionally accepted for publication in PLOS Neglected Tropical Diseases.

Before your manuscript can be formally accepted you will need to complete some formatting changes, which you will receive in a follow up email. A member of our team will be in touch with a set of requests.

Please note that your manuscript will not be scheduled for publication until you have made the required changes, so a swift response is appreciated.

IMPORTANT: The editorial review process is now complete. PLOS will only permit corrections to spelling, formatting or significant scientific errors from this point onwards. Requests for major changes, or any which affect the scientific understanding of your work, will cause delays to the publication date of your manuscript.

Should you, your institution's press office or the journal office choose to press release your paper, you will automatically be opted out of early publication. We ask that you notify us now if you or your institution is planning to press release the article. All press must be co-ordinated with PLOS.

Thank you again for supporting Open Access publishing; we are looking forward to publishing your work in PLOS Neglected Tropical Diseases.

Best regards,

Claudia Ida Brodskyn

Section Editor

PLOS Neglected Tropical Diseases

Claudia Brodskyn

Section Editor

PLOS Neglected Tropical Diseases

Shaden Kamhawi

co-Editor-in-Chief

PLOS Neglected Tropical Diseases

orcid.org/0000-0003-4304-636XX

Paul Brindley

co-Editor-in-Chief

PLOS Neglected Tropical Diseases

orcid.org/0000-0003-1765-0002

***********************************************************

# Editor report: Acceptance letter

**DOI:** 10.1371/journal.pntd.0014800.r004

**Authors:** Claudia Brodskyn, Anil Fastenau

Dear Dr Naipaul,

We are delighted to inform you that your manuscript, "Travel History and Occupation Among Diagnosed Cutaneous Leishmaniasis Cases in Guyana: A Multi-Site Retrospective Case Series, 2017–2021," has been formally accepted for publication in PLOS Neglected Tropical Diseases.

We have now passed your article onto the PLOS Production Department who will complete the rest of the publication process. All authors will receive a confirmation email upon publication.

The corresponding author will soon be receiving a typeset proof for review, to ensure errors have not been introduced during production. Please review the PDF proof of your manuscript carefully, as this is the last chance to correct any scientific or type-setting errors. Please note that major changes, or those which affect the scientific understanding of the work, will likely cause delays to the publication date of your manuscript. Note: Proofs for Front Matter articles (Editorial, Viewpoint, Symposium, Review, etc...) are generated on a different schedule and may not be made available as quickly.

Soon after your final files are uploaded, the early version of your manuscript will be published online unless you opted out of this process. The date of the early version will be your article's publication date. The final article will be published to the same URL, and all versions of the paper will be accessible to readers.

For Research Articles, you will receive an invoice from PLOS for your publication fee after your manuscript has reached the completed accept phase. If you receive an email requesting payment before acceptance or for any other service, this may be a phishing scheme. Learn how to identify phishing emails and protect your accounts at https://explore.plos.org/phishing.

Thank you again for supporting open-access publishing; we are looking forward to publishing your work in PLOS Neglected Tropical Diseases.

Best regards,

Shaden Kamhawi

co-Editor-in-Chief

PLOS Neglected Tropical Diseases

Paul Brindley

co-Editor-in-Chief

PLOS Neglected Tropical Diseases
